# Estimating the accumulative dose uncertainty for intracavitary and interstitial brachytherapy

**DOI:** 10.1186/s12938-021-00942-z

**Published:** 2021-10-18

**Authors:** Binbing Wang, Weibiao Hu, Guoping Shan, Xiaoxian Xu

**Affiliations:** 1 Department of Radiation Physics, Zhejiang Key Laboratory of Radiation Oncology, The Cancer Hospital of the University of Chinese Academy of Sciences (Zhejiang Cancer Hospital), Institute of Basic Medicine and Cancer (IBMC), Chinese Academy of Sciences, No. 1. East Banshan Road, Gongshu District, Hangzhou, 310022 Zhejiang China; 2 Taizhou Hospital of Zhejiang Province, Taizhou, 318000 Zhejiang China

**Keywords:** Accumulative dose, Intracavitary brachytherapy, Interstitial brachytherapy, Deformable registration

## Abstract

**Background:**

Image-guided adaptive brachytherapy shows the ability to deliver high doses to tumors while sparing normal tissues. However, interfraction dose delivery introduces uncertainties to high dose estimation, which relates to normal tissue toxicity. The purpose of this study was to investigate the high-dose regions of two applicator approaches in brachytherapy.

**Method:**

For 32 cervical cancer patients, the CT images from each fraction were wrapped to a reference image, and the displacement vector field (DVF) was calculated with a hybrid intensity-based deformable registration algorithm. The fractional dose was then accumulated to calculate the position and the overlap of high dose (D2cc) during multiple fractions.

**Result:**

The overall Dice similarity coefficient (DSC) of the deformation algorithm for the bladder and the rectum was (0.97 and 0.91). No significant difference was observed between the two applicators. However, the location of the intracavitary brachytherapy (ICBT) high-dose region was relatively concentrated. The overlap volume of bladder and rectum D2cc was 0.42 and 0.71, respectively, which was higher than that of interstitial brachytherapy (ISBT) (0.26 and 0.31). The cumulative dose was overestimated in ISBT cases when using the GEC-recommended method. The ratio of bladder and rectum D2cc to the GEC method was 0.99 and 1, respectively, which was higher than that of the ISBT method (0.96 and 0.94).

**Conclusion:**

High-dose regions for brachytherapy based on different applicator types were different. The 3D-printed ICBT has better high-dose region consistency than freehand ISBT and hence is more predictable.

## Background

Image-guided adaptive brachytherapy (IGBT) has been widely implemented to increase treatment accuracy and improve survival rates. Due to the use of high fractional doses and steeper dose gradients, evaluating the cumulative dose–volume for tumor targets and organs at risk (OARs) from several fractions is regarded as essential in brachytherapy. Previous studies have reported that higher dose–volume histogram (DVH) parameter values for the bladder and rectum are associated with an increased risk of radiation toxicity [1, 2]. Hence, the treatment plan should meet the requirement for the minimum dose value in the 2 cc most irradiated bladder and rectum value, defined as D2cc, which is recommended by The Group Européen de Curiethérapie-European Society for Radiotherapy and Oncology (GEC-ESTRO) [3]. However, D2cc reports on the delivery of high doses to organs and provides no information on the spatial distribution of these high-dose regions. In addition, this evaluation strategy does not consider areas that may not even overlap among the fractions.

Deformable image registration (DIR) searches for nonlinear spatial transformation relationships. It is used in medical image processing during atlas-based segmentation [4, 5] and multimodal image fusion [6, 7]. Dose accumulation is an application of DIR during radiotherapy [8, 9]. The DVF calculated between fixed CT and each moving CT image assists in mapping the fixed image by the radiation dose in moving CT images**.** Related studies have included the use of cone-beam CT (CBCT) in external beam radiotherapy (EBRT) to evaluate the actual doses received by the tumor targets and OAR on the day of treatment [10, 11]. The doses of EBRT and brachytherapy (BT) were accumulated [12], and the cumulative dose between BT fractions was calculated [13].

Unfortunately, the accuracy of dose deformation highly depends on image deformation. The uncertainty of DIR transmits with DVF to the stage of cumulative dose deformation, affecting the final dose distribution, and other uncertainties are introduced into it. These might occur due to the uncertainties obtained from delineation and the DIR algorithm in BT [14, 15]. Thor et al. reported differences in magnitude in the bladder generalized equivalent uniform dose (gEUD) with a median dose of 47 Gy versus 57 Gy for two DIR algorithms [16]. Andersen et al. reported that the use of a larger structure as a reference frame for dose accumulation may result in an increased value of the high-dose region when mapped to a smaller structure [17]. Moreover, uncertainties might also occur due to different types of applicators. Insertion operations inevitably lead to differences even if a standard mode of applicator is used with external fixation. Lang et al. compared the total dose (EBRT + BT) parameter difference between one insertion with two fractions and the individual insertion of each fraction. They observed differences in dose distributions among different treatment fractions, making it difficult to calculate the cumulative dose. A variation of 22% in the bladder and sigmoid D2cc dose has been reported when tandem and ring applicators are used [18]. Andersen et al. reported significant differences in dose distribution after using intracavitary/interstitial (IC/IS) BT, and multiple fractional studies might be associated with more inconsistent dose distributions [19]. For ISBT, due to freehand insertion, the dose distribution between different fractions can vary. The location of high-dose D2cc is closely related to the radiotoxicity of normal tissues. The unpredictable location causes difficulty for assessment. Recently, promising advancements have been reported using 3D-printed cylinder applicators, which can provide better organ sparing [20–22]. The 3D-printed cylinder applicator uses a relatively fixed catheter position, size, and shape. It is expected to generate a consistent dose distribution between the fractions, which might in turn reduce the uncertainty in treatment.

It is important to critically evaluate interfractional high doses both quantitatively and locationally, which is a crucial parameter to predict the probability of brachytherapy normal tissue complications. From the treatment planning viewpoint, different imaging, contouring, and source distributions result in different dose distributions. The use of appropriate evaluation methods for different insertion approaches has clinical value. As stated above, the interfractional dose variation has been investigated for some applicators. However, the performance of 3D-printed cylinders has not yet been reported. In this study, we aim to compare the accumulated dose for two insertion approaches, which enables us to clarify whether the position change of the high-dose region depends on the difference in the dose distribution of each fraction or changes in the organ position and filling. We used a 3D-printed cylinder ICBT applicator to make the source distribution consistent between fractions and compared it with ISBT. The GEC-recommended high-dose region calculation method was chosen as the baseline. The DIR method was then used to calculate the position of the high-dose regions, and the difference in the cumulative dose calculation of the baseline method was compared.

## Results

### (A) DIR performance

A total of 97 deformations were reviewed in this study. The number of cases with a bladder volume > 400 cc or a rectal volume > 100 cc remained small. In Table 1, the deformation volume difference Diff_Vol_ was presented as the ratio of the volume between the fixed and moving images, which is an indicator of the feasible range of DIR. Previous works recommended that it should be less than 2; otherwise, DIR will cause unexpected results [23]. The Diff_Vol_ of the bladder for the same patient was 0.72–1.53 for ICBT and 0.64–1.67 for ISBT, showing no significant difference (*p* = 0.24). The Diff_Vol_ of the rectum between the ICBT group and ISBT group was 0.43–1.51 and 0.67–1.31, respectively, without any significant difference (*p* = 0.19). Both ICBT and ISBT had close average DSC scores. ISBT had DSCs of 0.98 (range 0.76–0.99) for the bladder and 0.97 (range 0.8–0.98) for the rectum compared to ICBT of 0.97 (range 0.81–0.99) for the bladder and 0.95 (range 0.87–1) for the rectum, with no difference.

**Table 1:** Deformable image registration outcomes of two different applicators in this study

	Bladder			Rectum		
	ICBT (*n* = 50)	ISBT (*n* = 47)	*p*	ICBT (*n* = 50)	ISBT (*n* = 47)	*p*
Diff_Vol_	0.72–1.53	0.64–1.67	0.24	0.43–1.51	0.67–1.31	0.19
Dice	0.97 (0.87–0.99)	0.98 (0.81–1)	0.17	0.95 (0.8–0.98)	0.97 (0.87–1)	0.01
≥ 0.9, *n* (%)	48 (96)	45 (95.7)		45 (90)	45 (95.7)	
*V*_ol_ (cc)	0.42 ± 0.40	0.28 ± 0.25	< 0.01	0.75 ± 0.46	0.31 ± 0.32	< 0.01
*V*_all_ (cc)	4.43 ± 1.69	5.06 ± 2.44	< 0.01	4.26 ± 1.52	5.90 ± 2.15	< 0.01
D2cc (DIR/GEC)	0.99 (0.94–1.05)	0.96 (0.91–1.07)	< 0.01	1.00 (0.96–1.08)	0.94 (0.91–1.06)	< 0.01
Overestimated, *n* (%)	5 (10)	6 (12.8)		6 (8)	1 (10.6)	

### (B) High-dose region distribution

**Fig. 1:**
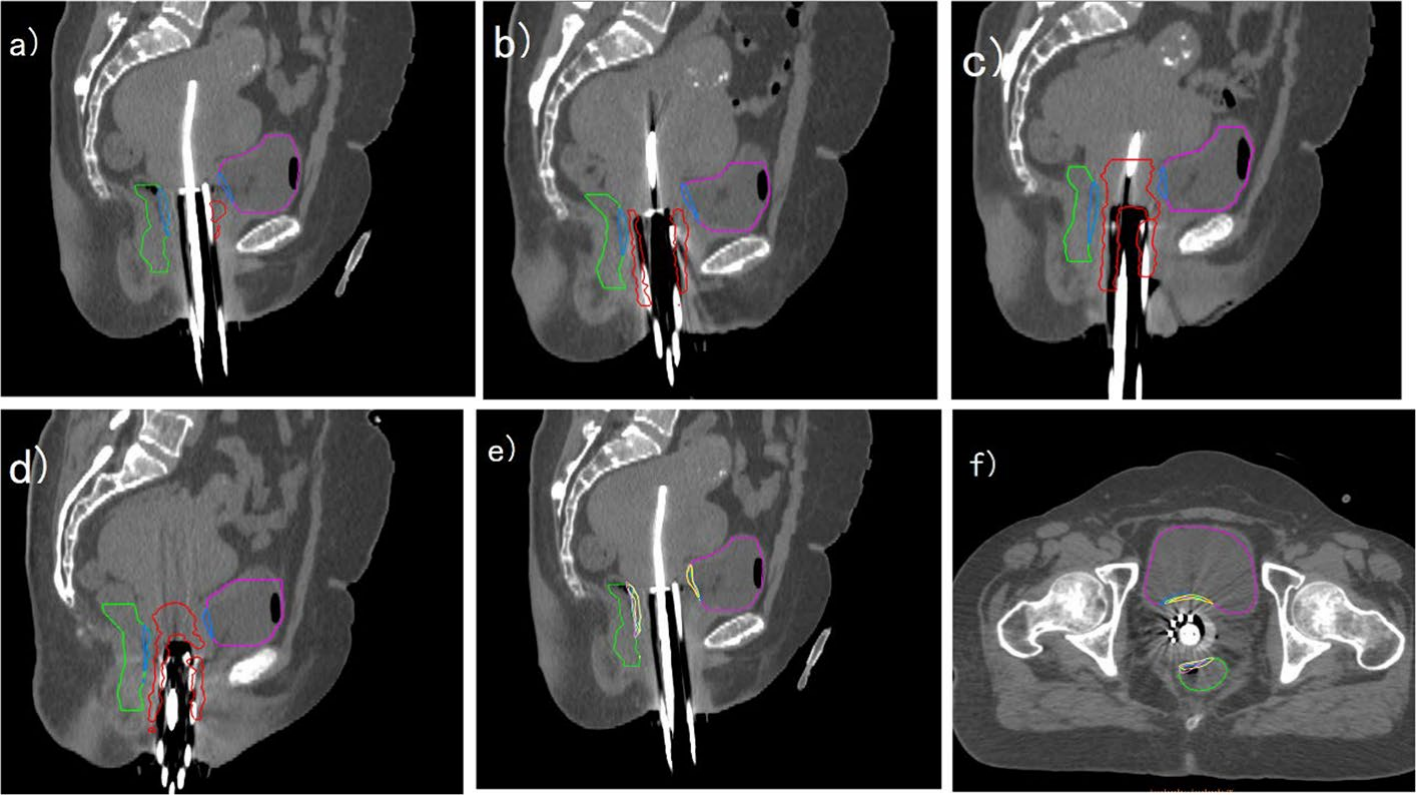
A patient with accumulative dose estimation of ICBT. The purple contour represents the bladder and the green contour represents the rectum. The high-dose area is distributed around the applicator. The 2 cc volume is distributed near the cervix of each fraction is shown in **a**–**d**. The 2 cc volume after deformation is distributed in either the posterior wall of the bladder or the anterior wall of the rectum as shown in **e** and **f**

**Fig. 2:**
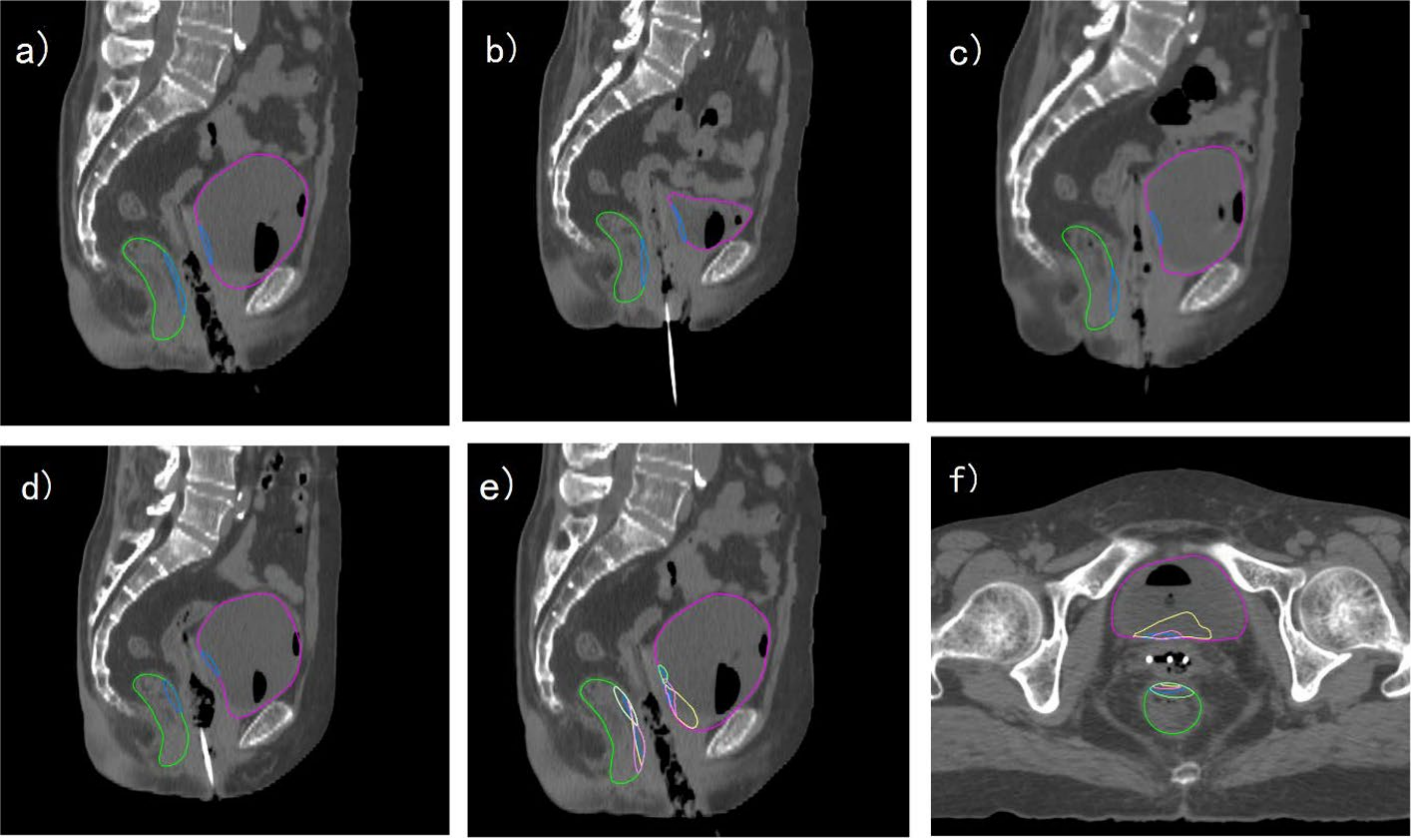
A patient with accumulative dose estimation of ISBT approach. The purple contour represents the bladder and the green contour represents the rectum. The D2cc region of each fraction is shown in **a**–**d**. The 2 cc volume distribution is different between fractions. D2cc region is distributed dispersed in deformed image shown in **e** and **f**

According to the observation of each fraction, the high-dose region (D2cc) distribution of the two types of applicators was different. Figure 1 shows 2 cc regions changed in each fraction of a ICBT case. The D2cc regions were distributed near the cervix of each fraction. From the sagittal view, the 2 cc regions on bladder and rectum were in the same position. Although the bladder and rectal volume were different between multi-fractions, the 2 cc positions after deformation were still overlapped in both sagittal view and axial view. Rectal fillings were consistent between fractions shown in Fig. 2 but the 2 cc position was more dispersed in the longitudinal distribution than ICBT. Therefore, the overlap area after deformation is also smaller than ICBT.

The distribution of D2cc volume is also summarized for both plans in Table 1. For 13 cases of ISBT, the total volume *V*_all_ of the bladder D2cc after deformation was found to be 5.06 ± 2.44 cc, and the overlap volume *V*_ol_ was 0.28 ± 0.25 cc. The rectal D2cc *V*_all_ was 5.90 ± 2.15 cc, and *V*_ol_ was 0.31 ± 0.32 cc. In 4 cases, the bladder D2cc volume did not overlap. Meanwhile, the rectal D2cc volume of 3 patients did not overlap. For ICBT plans, the D2cc *V*_all_ for the bladder was 4.43 ± 1.69 cc, and *V*_ol_ was 0.42 ± 0.40 cc. Only 1 case of bladder D2cc and 1 case of rectal D2cc were observed, with no regions of overlap between the fractions.

### (C) Accumulated dose via GEC method

The implementation of different applicator types can cause differences in the total dose estimation. The GEC method was chosen as the baseline value. A ratio of D2cc (DIR)/D (GEC) was defined to demonstrate the accumulated DIR dose change from baseline. As assessed, the ICBT with the 3D-printed applicator also showed good dose value consistency both in interfraction and between different cases. Table 1 shows that the average D2cc (DIR)/D (GEC) values in the bladder and rectum were 0.99 (0.94–1.05) and 1.00 (0.96–1.08), respectively. However, the difference between the DIR cumulated D2cc and GEC methods for ISBT was large. The average DIR D2cc for ISBT applicator insertion was lower than that of the GEC D2cc addition, with values of 0.96 (0.91–1.07) and 0.94 (0.91–1.06) in the bladder and the rectum, respectively.

## Discussion

In this study, the 2 cc high-dose region obtained by the two applicator approaches, the 3D-printed cylinder ICBT and the ISBT with needle implantation, was compared in estimating the cumulative dose with or without DIR. The 3D-printed cylinder ICBT approach generates a consistent high-dose region location during the fraction that can be predicted accurately by GEC accumulation. In contrast, the GEC method failed to evaluate the total dose for the ISBT approach. Andersen et al. reported that simple DVH parameter addition overestimated bladder D2cc by 1.5% with ICBT and ISBT. Dose deviations of greater than 5% relative to DIR were found in 2% of patients [17]. Kobayashi observed more significant differences in bladder D2cc of 2.8% when all fractions were summed up [24]. Molton et al. compared rectal dose accumulation using DIR-based addition and DVH-based addition in the context of combining EBRT/HDR-BT prostate cancer treatment with that of metal after-loading catheters. They found that the D2cc from DVH addition was 3.5% smaller than those obtained from DIR addition [8]. Our results were more significant than their findings, with a dose variation of approximately 4% for bladder dose addition and 6% for rectum dose addition in ISBT plans.

Deformable registration-based accumulation was also employed to evaluate the 2 cc high-dose region by the two applicator approaches. The propagation of the deformation inside the contours, which is very dependent on the relative volume, becomes critical, making the accumulation inaccurate. In this work, a median volume DIR algorithm strategy was used. The use of a volume control protocol during CT scanning also avoided relative volume dependence. Thörnqvist et al. evaluated the performance of the DIR algorithm based on inaccuracies. They observed that a reduction in the bladder volume compared to the fixed image led to a decrease in the DSC. Additionally, the DSC score decreased rapidly when the relative volumes were larger than 1.4 [23]. Although the DSC metric accounted for the whole contour, we found that discrepancies in DSC had a limited impact on bladder DVH metrics because it is usually observed in low-dose areas. However, unexpected deformation tends to appear in the rectum even for cases with closer volume differences. Discrepancies in dose deformation were more pronounced in most of the slices. For that reason, efforts are going on to improve deformation quality by image preprocessing approaches. Flower et al. reported increasing uncertainties in the pelvis DIR due to the effects of gas, low image contrast, and large deformations in the rectum [13]. Ryckman et al. found that the optimization goal of the DIR process can be achieved by assigning the CT Hounsfield values to each ROI individually to create higher performance between the ROIs [12]. Thus, it may be difficult to predict when inaccurate rectal deformation is performed, and sometimes a poor result may be encountered.

We also defined the overlap volume *V*_ol_ and combined volume *V*_all_ to further estimate the localization of high-dose regions in the fractions. Andersen et al. evaluated the variation in the D2cc position between the two fractions with combined IC/IS insertion. They found that the D2cc position categorized as “similarly” has a significant difference in the total dose when compared to those categorized as “dissimilarly” based on visual inspection [17]. For this reason, it is important to evaluate the localization of high-dose regions. The interfractional 2 cc location showed an apparent discrepancy between the ICBT and ISBT approaches due to dwelling time, and the position setting was loaded for each fraction. In addition, the usage of IC applicators improved reproducibility, which can be predicted more accurately by the GEC method.

There are a few limitations in this study. The results can be different due to the use of different deformation algorithms [25]. Research has indicated that there are large discrepancies in the shifts by directly comparing the performance of various algorithms [26]. Jamema et al. compared two DIR algorithms and reported that the DIR based on different DIR algorithms might cause a systematic underestimation of dose, leading to large differences between deformable dose accumulation and direct addition [14]. In this study, we chose ANACONDA as the DIR algorithm but did not compare it with other algorithms. Although a discrepancy was observed in the upper boundary of the bladder, this study focused on the D2cc location in the high-dose region concentrated in the lower bladder, where the discrepancy had little effect on the final results.

## Conclusions

The fractional dose accumulation for OAR as recommended by the GYN GEC ESTRO working group is essential for treatment planning of IGBT. In this study, high-dose region distributions of 3D-printed ICBT and ISBT approaches between different fractions were observed. The total volume *V*_all_ of the highest 2 cc in the bladder was 4.43 ± 1.69 cc with ICBT and 5.06 ± 2.44 cc with ISBT. The overlap volume *V*_ol_ also showed the same trends, which were 0.42 ± 0.40 cc with ICBT and 0.28 ± 0.25 cc with ISBT. The average D2cc (DIR)/D (GEC) value in the bladder was 0.99 (0.94–1.05) with ICBT and 0.96 (0.91–1.07) with ISBT. We concluded that 3D-printed ICBT has better high-dose region consistency between fractions than freehand ISBT. We also verified that the GEC-recommended method predicted better ICBT cumulative dose calculations than ISBT by the method of deformation dose accumulation. In future work, we will validate the accuracy of DIR between different media and large volume deformation with various deformation algorithms.

## Methods

### Patient selection

A total of 32 patients with cervical cancer from February 2016 to October 2019 were selected for this study. Nineteen patients received a 3D-printed cylinder applicator for uterine and vaginal deliveries. Thirteen patients received freehand steel interstitial needle implantation for the uterine body and parauterine region. Patients treated with other applicator insertions were excluded from this study. This study was approved by the research ethics board, and written informed consent was acquired from all the patients. CT scanning was performed for each fraction of BT with a 2.5 mm slice thickness. All patients were treated with 3–5 fractional BT plans. High-risk clinical target volume (HR-CTV) and OARs (bladder, rectum, and sigmoid) were delineated according to the GEC-ESTRO Working Group recommendations [3]. A prescription of 40 Gy_EQD2_(*α*/*β* = 10 Gy) was given to HR-CTV D90 and the D2cc dose limitations for bladder was 40 Gy_EQD2_(*α*/*β* = 3 Gy) and 30 Gy_EQD2_(*α*/*β* = 3 Gy) for rectum.

### 3D-printed cylinder

**Fig. 3:**
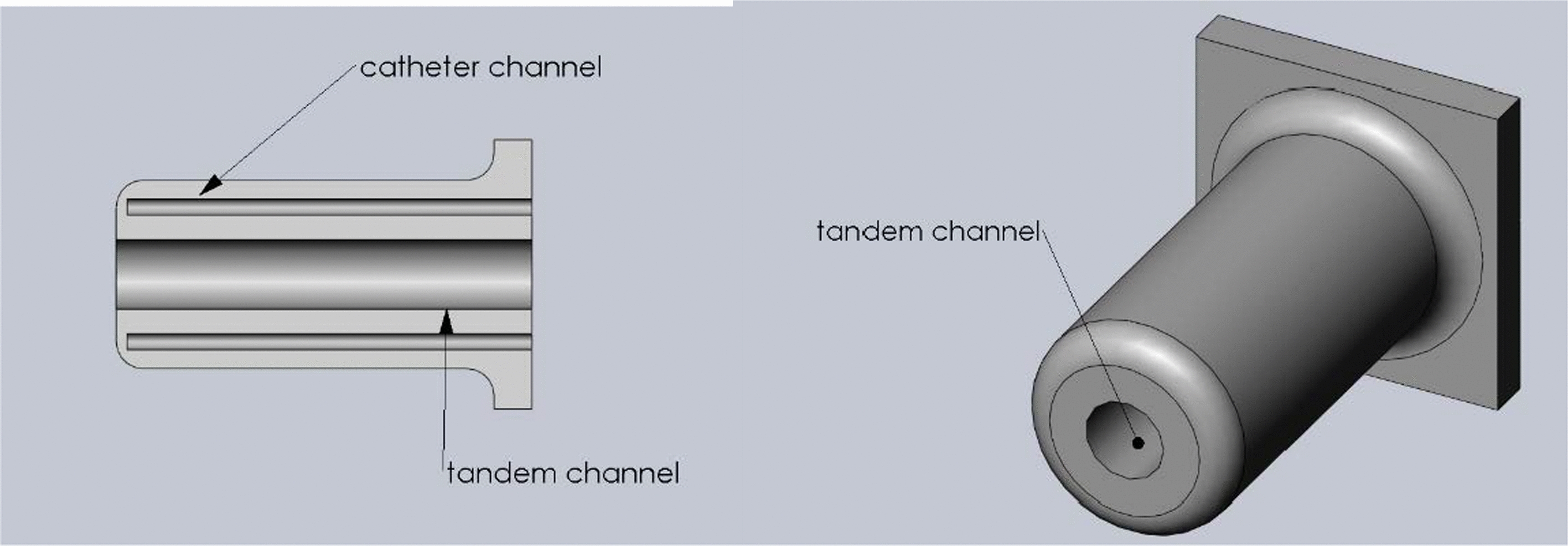
The prototype of 3D-printed ICBT applicator

Experience with 3D-printed applicators includes easy handling and providing repeatedly precise needle guidance by the similarity of the obtained physical doses [27]. In this study, a 3D-printed cylinder applicator was modeled with a diameter of 35 mm and 180 mm in length. The applicator had multiple channels for catheters and one central channel for tandem insertion, as illustrated in Fig. 3. The applicator was 3D printed using a fused deposition modeling printer (MBot 3D Grid II + , Magicfirm) in biocompatible material (Stratasys Ltd. Eden Prairie, MN, USA). The 3D-printed applicator was sterilized by our hygiene unit before clinical use was initiated. The catheters were then placed along the surface of the vaginal cylinder to modulate the desired dose distribution. For tumors with irregular shapes, peripheral channels can provide asymmetric dose coverage of the target while maintaining normal tissue constraints.

### DIR procedure

**Fig. 4:**
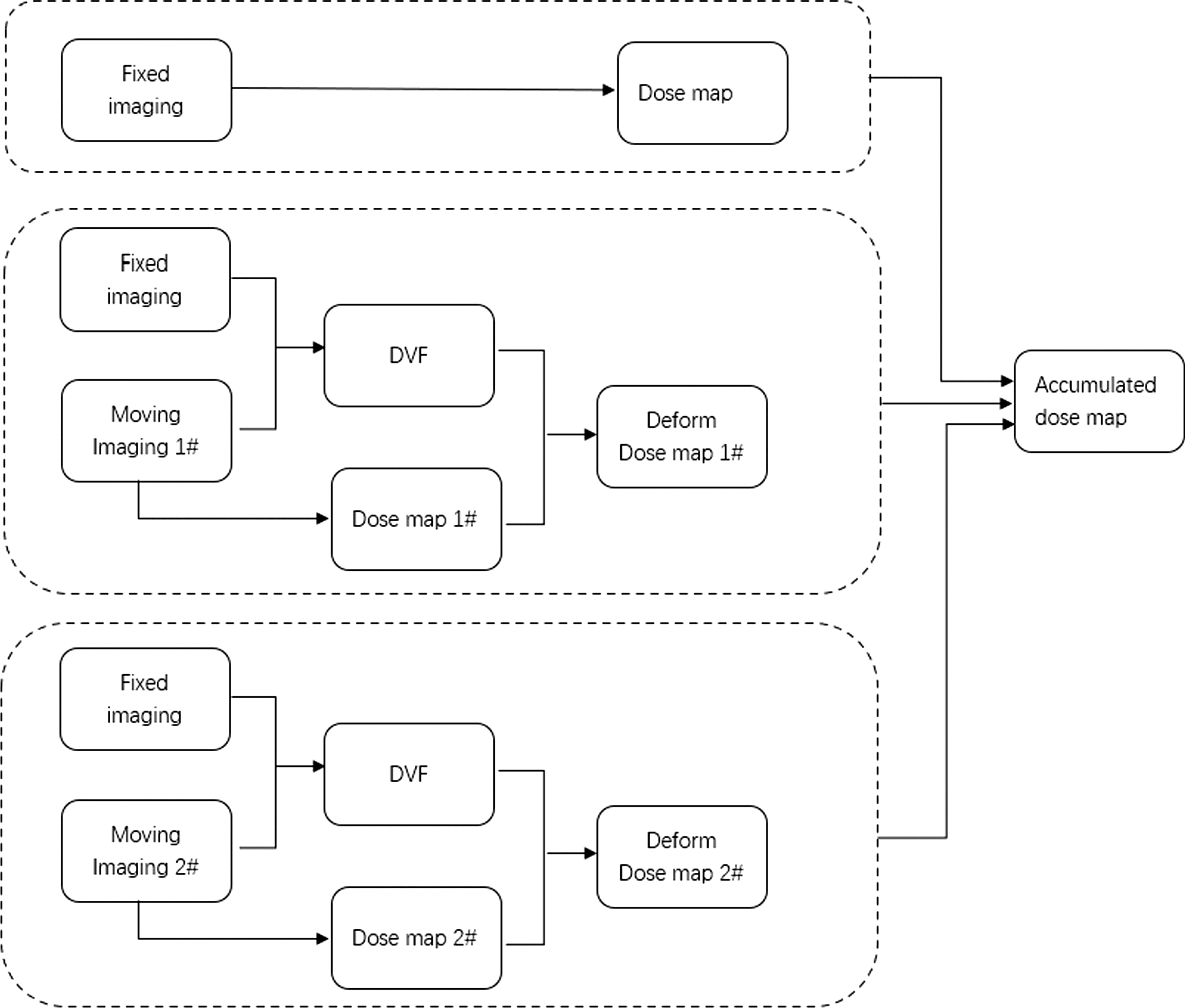
The deformation process of multi-fraction dose accumulation

Previous studies have reported that the deformation algorithm does not work well for registering large deformation images due to the linearization of elasticity [16]. The selection of the minimum or maximum volume leads to large deformation, which could cause deformation failure. Moreover, when deformation propagates a smaller volume of the dose to a larger volume, the high-dose region is enlarged accordingly, which can make the resultant dose variation larger. In this study, CT images with median bladder/rectum volume were selected as fixed images. Rigid registration was performed using mutual information before deformation. The fixed image was the reference image, and the moving image was wrapped to align it. The deformation process is demonstrated in Fig. 4. An intensity-based DIR algorithm (ANACONDA) was used in this study [28, 29]. ANACONDA is a hybrid algorithm that combines image information with anatomical information as provided by contoured image sets. The region of interest (ROI) was contoured before deformation, and then the DVF between the fixed image and the moving image was calculated according to the boundary of the ROI and the gray level of the image. Due to the combination of using both image similarity and anatomical information, ANACONDA is capable of handling low contrast regions of the pelvic region [28]. The objective function of ANACONDA is defined as:

$$f\left(v\right)=\alpha C\left(v\right)+\left(\beta H\left(v\right)+\gamma S\left(v\right)\right)+\delta D\left(v\right),$$

where $C\left(v\right)$, $\left(H\left(v\right)+S\left(v\right)\right)$, and $D(v)$ are the image similarity measure term, regularization term and controlling structure term, respectively. Unlike the model-based DIR algorithm, which describes biomechanical materials using finite element modeling, ANACONDA penalizes large shape deviations of ROIs using $\gamma S\left(v\right)$. Therefore, it has the flexibility to handle large anatomical changes due to differences in bladder and rectum filling in a reasonable range.

In this study, the bladder, rectum, and external body were taken as the focus ROIs. The Dice similarity coefficient is a statistic used to gauge the similarity of two samples [30]. It has been widely employed as a similarity metric of two contours in image segmentation. In this study, DSC was defined as the degree of coincidence of the contour of the moving image and its corresponding contour on the fixed image. If the contours on the moving image were completely coincident, then the DSC value was equal to 1. DSC was defined as:

$$\mathrm{DSC}=\frac{2\left({V}_{\mathrm{fixed}}\cap {V}_{\mathrm{moving}}\right)}{{V}_{\mathrm{fixed}}+{V}_{\mathrm{moving}}}.$$

The DSC value was obtained by Boolean operation on the contour of the corresponding organ.

Dose mapping was obtained from the DVF as a cumulative dose of fraction. Due to differences in the D2cc dose values in each case, it is hard to compare the dose directly between cases. Therefore, this study defined the dose ratio D2cc (DIR)/D (GEC). Here, D2cc (GEC) was used as the calculation method recommended by GEC-ESTRO, and D2cc (DIR) was obtained from the accumulation dose. To address the location of D2cc contours, the overlap volume *V*_ol_ and combined volume *V*_all_ of D2cc contours were defined as the total volume that was propagated from the moving images.

Comparisons between the volume and dose values of the two applicator approaches were analyzed using the independent sample *t*-test. All statistical tests were two-tailed, with a threshold for statistical significance of a *p*-value < 0.05. Statistical analyses were carried out using SPSS version 25.0 software.

## Abbreviations

EBRT: External beam radiotherapy
BT: Brachytherapy
HR-CTV: Clinical target volume
ICBT: Intracavitary brachytherapy
ISBT: Interstitial brachytherapy
DVF: Displacement vector field
OAR: Organ at risk
gEUD: Generalized equivalent uniform dose
GEC-ESTRO: The Group Européen de Curiethérapie-European Society for Radiotherapy and Oncology
DIR: Deformable image registration

## Declarations

### Availability of data and materials

The datasets used and/or analyzed during the present study are available from the corresponding author upon reasonable request.
